# NZTD - The New Zealand Trait Database for shallow-water marine benthic invertebrates

**DOI:** 10.1038/s41597-023-02414-6

**Published:** 2023-07-29

**Authors:** Orlando Lam-Gordillo, Andrew M. Lohrer, Judi Hewitt, Sabine Dittmann

**Affiliations:** 1 National Institute of Water and Atmospheric Research, Hamilton, New Zealand; 2 College of Science and Engineering, Flinders University, Adelaide, Australia; 3 Department of Statistics, University of Auckland, Auckland, New Zealand

**Keywords:** Ecosystem ecology, Marine biology

## Abstract

Macrobenthic traits, for example feeding mode, life history, morphology, are increasingly used for determining responses of macrobenthic fauna to environmental change and influences on ecosystem functioning. Yet, trait information is scarce or non-existent in several parts of the world, such as New Zealand. This deficit makes collecting trait data a difficult and time-consuming task, limiting its potential use in trait-based assessments. Here, we present the New Zealand Trait Database (NZTD) for marine benthic invertebrates, the first comprehensive assessment of macrobenthic traits in New Zealand. The NZTD provides trait information for more than 700 macrobenthic taxa, categorised by 18 traits and 77 trait modalities. The NZTD includes five freely downloadable datasets, (1) the macrobenthic trait dataset, with outcomes from a fuzzy coding procedure, (2) the trait source information, (3) the references by taxa, (4) the full references list, and (5) the full taxa list used in the NZTD. Establishing the NZTD closes the trait knowledge gap in New Zealand and facilitates future research applying trait-based approaches to New Zealand’s coastal macrofauna.

## Background & Summary

Traits are properties of organisms that can be measured, usually at the individual organism level and used comparatively across species^1–3^. Traits are generally assigned based on feeding mode, life history, morphology, physiology, and behavioural characteristics of species^4–6^. In recent decades, the use of trait-based analyses has expanded, advancing our understanding of marine ecosystem functioning^3,7–9^ and how organisms are responding to environmental change (e.g. environmental gradients, anthropogenic disturbances, and climate change)^3,6,10,11^.

One challenge in applying trait-based approaches is the lack of knowledge and availability of species trait information. Macrobenthic fauna, defined as organisms retained on 0.5 mm mesh size, usually include large numbers of invertebrate taxa (e.g., polychaetes, crustaceans and molluscs), many of which are rare, small, cryptic, and inadequately studied. Therefore, gathering trait information from the literature or biological collections can be difficult and time-consuming^3,6,12,13^. Worldwide, several efforts have been made to alleviate the lack of macrobenthic trait information, for example establishing trait databases for the Arctic^14^, Southern Australia^13^, and Northwest Europe^12^, in addition to well-established online databases (e.g. MArLIN^15^, WoRMS^16^), and taxa-specific trait databases (e.g. Polytraits^17^). Despite these efforts, information about traits of macrobenthic fauna in several regions around the World is scarce or non-existent^3^, limiting the use of trait-based assessments that could assist conservation and management actions and increase understanding of the functioning of benthic ecosystems in those regions.

This article presents the New Zealand Trait Database (NZTD), aiming to (i) close knowledge gaps on macrobenthic trait information, and (ii) advance trait-based approaches for New Zealand. The NZTD is an open access database that followed the structure and traits presented in previous research^7,13,14^ for easy comparability and sharing among researchers. The NZTD provides trait information for more than 700 taxa. This is the first comprehensive assessment focusing on traits of marine macrobenthic fauna of New Zealand. Our aim was to provide a reliable source of macrobenthic trait information and facilitate further research using trait-based perspectives in New Zealand marine waters.

## Methods

### Data acquisition

**Fig. 1:**
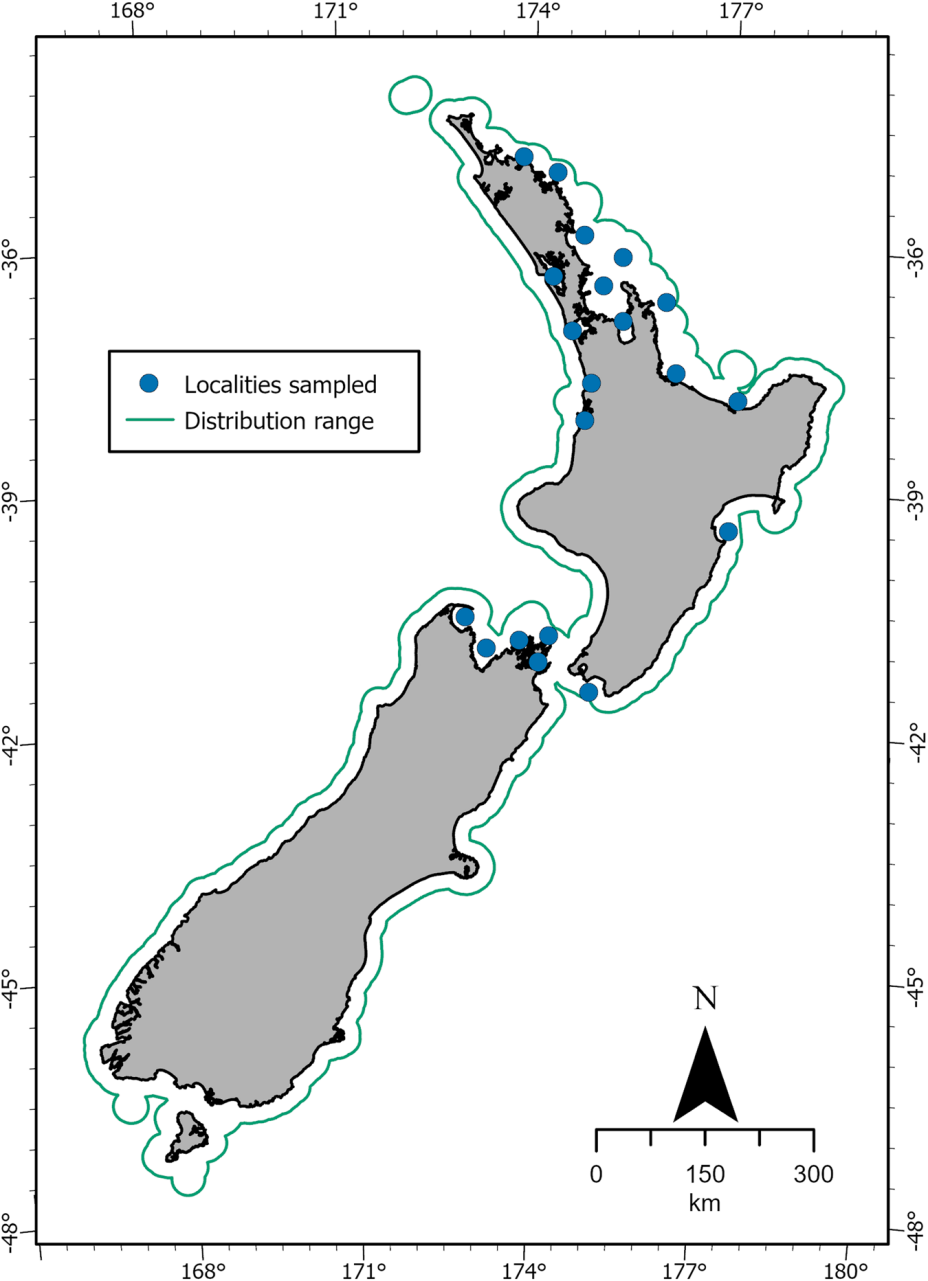
Localities sampled across New Zealand from where information about macrobenthic fauna was retrieved for the assessment of macrobenthic traits used for the NZTD. Blue dots show the main regions sampled over time, while the green solid line shows the overall extent of macrobenthic distribution covered (ranging from 0 to 20 m water depth) in the database.

Macrobenthic data (see full macrobenthic taxa list^18^) were compiled from previous published research on macrobenthic fauna^19–33^ carried out by the authors in 20 different localities within New Zealand (Fig. 1, see also technical validation section). Briefly, sediment samples for assessing macrobenthic fauna were collected using a hand-held PVC corer (70 mm diameter) pushed into the sediment up to 10 cm depth. Macrobenthic fauna samples were then sieved through a 0.5 mm mesh size and preserved in 70% isopropanol. Benthic macrofauna were then sorted, identified to the lowest possible taxonomic level, and counted. The dataset encompasses a mix of records from soft sediments of New Zealand, including muddy-to-sandy substrates, vegetated (seagrass, mangrove) and unvegetated (microphytobenthos only) coastal ecosystems in estuaries and on the exposed coast.

### Selection of traits

The traits presented in the NZTD were selected based on a published global review^3^ describing their relevance for the assessment of ecosystem functioning and identified as the most commonly used traits for assessing macrobenthic fauna^3^. Our selection also considered traits that could be compared across studies and geographical areas, i.e., are applicable to most benthic assemblages^6,7,13^. In total, 18 traits and 77 trait modalities allocated across four different subject areas (e.g. biology, habitat, life-history and larval type)^13^ were assessed (Tables 1–4).

**Table 1:** Summary information about the 18 traits and 77 trait-modalities included in The New Zealand Trait Database (NZTD) of marine benthic invertebrates (Modified from^13^).

Subject area	Traits	Modalities	Definition	Function and processes	Reference
Biology	Bioturbation	Biodiffusor	Transport processes and modification of sediments carried out by organisms that directly or indirectly affect sediment composition.	Nutrient cycling, sediment reworking, organic matter re-generation, influence on biogeochemistry.	^6,10,13,42–44^
		Bioirrigator			
		No bioturbation			
		Surface modifier			
	Body size	Large (>20 mm)	Maximum body size as adult.	Influence on productivity, habitat facilitation, sediment reworking, oxygen consumption.	^6,7,10,44^
		Medium (5–20 mm)			
		Small (0.5–5 mm)			
	Degree of attachment	None	Organism ability of attach themselves to a substratum.	Influence on metabolic production, trophic support, habitat facilitation.	^5,44^
		Permanent			
	Feeding mode	Deposit feeder	The mode of food acquisition.	Nutrient cycling, resource utilization and facilitation, species demographic control, trophic support.	^6,7,10,44,45^
		Filter/suspension			
		Grazer/scraper			
		Omnivore			
		Predator			
		Scavenger/opportunist			
		Sub-surface deposit feeder			
	Living habit	Attached	Organism mode living as an adult.	Nutrient cycling, sediment transport, dispersal, habitat creation and facilitation.	^6,44,46^
		Burrower			
		Free living/Surface crawler			
		Parasite/Commensal			
		Tube dwelling

**Table 2:** Continuation of the summary information about the 18 traits and 77 trait-modalities included in The New Zealand Trait Database (NZTD) of marine benthic invertebrates (Modified from^13^).

Subject area	Traits	Modalities	Definition	Function and processes	Reference
Biology	Mobility	Limited	Degree of movement.	Nutrient cycling, sediment reworking, trophic support, food source.	^6,7,44^
		Mobile			
		Sessile/attached			
	Morphology	Irregular	External features and overall shape of an adult organism.	Sensitivity, food source, habitat facilitation, survival to disturbances, sediment reworking.	^6,10,44^
		Round/Globulose			
		Streamlined			
		Vermiform			
	Movement method	Burrower	Organism type of movement as an adult.	Nutrient cycling, sediment transport, dispersal, recolonization, migration, ability to escape predation.	^6,10,44^
		Crawler			
		None			
		Swimmer			
	Rigidity	Hard exoskeleton	External structural robustness of an adult organism.	Sensitivity, food source, habitat facilitation, survival to disturbances, sediment reworking.	^6,10,44^
		Hard shell			
		Rigid/Rigid tubes			
		Soft/Fragile			
	Structural fragility	Very fragile/Highly breakable	External flexibility of an adult organism.	Sensitivity to physical damage.	^6,10,44^
		Fragile/Limited flexibility			
		Very strong/Extremely flexible

**Table 3:** Continuation of the summary information about the 18 traits and 77 trait-modalities included in The New Zealand Trait Database (NZTD) of marine benthic invertebrates (Modified from^13^).

Subject area	Traits	Modalities	Definition	Function and processes	Reference
Habitat	Sediment transport	Conveyor belt	Transport processes of sediments carried out by organisms that directly or indirectly affect sediment composition.	Nutrient cycling, sediment reworking, organic matter re-generation, influence on biogeochemistry.	^6,10,16,19–21^
		Diffusive mixing			
		No transport			
		Reverse conveyor belt			
		Surface deposition			
	Sediment position	Attached	Organism relative position on the sediment.	Nutrient cycling, sediment transport, habitat creation and facilitation.	^6,7,10,44,46^
		Bentho-pelagic			
		Epibenthic			
		Crevices, stones, shells			
		Deeper than 3 cm			
		Surface shallow <3 cm			
	Sediment stabiliser	Destabiliser	Modification of sediments carried out by organisms.	Nutrient cycling, sediment transport, habitat creation and facilitation.	^10,13,42^
		None			
		Stabiliser			
	Sediment surface topography	Mound	Modification to the furface of sediments carried out by organisms.	Habitat creation and facilitation, sediment complexity, trophic support.	^35,42,44^
		None			
		Permanent burrow			
		Simple hole or pit			
		Trampling			
		Trough			
		Tube structure

**Table 4:** Continuation of the summary information about the 18 traits and 77 trait-modalities included in The New Zealand Trait Database (NZTD) of marine benthic invertebrates (Modified from^13^).

Subject area	Traits	Modalities	Definition	Function and processes	Reference
Larval	Larval type	Pelagic -planktotrophic	Larval type and feeding mode.	Food source, ability of species dispersal, influence in nutrient cycling.	^6,44,46^
		Pelagic lecthrotophic			
		Benthic			
		Brooder/Direct developer			
		No larvae			
Life-history	Life span	<1 year	Organism maximum life span as an adult.	Community dynamics, resilience of organisms, reproduction, productivity.	^6,10,44,46^
		1–3 years			
		3–10 years			
	Reproductive frequency	Iteroparous	Times that the organism reproduce over time.	Demographic resilience, population stock.	^6,10^
		Semelparous			
		Semi-continuous			
	Reproductive technique	Asexual	The mode organism reproduces, mechanism of fertilization and propagules released.	Species dispersal, carbon transport, demographic resilience.	^6,7,10^
		Sexual, pelagic shed eggs			
		Sexual, benthic shed eggs			
		Sexual, encapsulation (gelatinous mass)			
		Sexual, ovigerous, broad eggs			
		Sexual direct development			

### Trait allocation

Trait information was retrieved from different published primary literature (e.g., peer reviewed journal articles, books, thesis), secondary literature (e.g. online resources, reports), and expert knowledge (see full trait source database^34^), depending on the availability of information for each taxon. We categorised the sources of the trait information into four categories based on origin: (1) New Zealand literature, (2) Australian literature, (3) Overseas literature, and (4) Expert knowledge/Online resources. We followed the approach used in the South Australian Trait Database^13^ and allocated the trait information based on the available information for each taxon. When trait information of a particular taxon was missing, trait information from the closest phylogenetic taxon was used. For example, if no trait information was available at Species level, trait information was used from another species within the same Genus; if information was unavailable at Genus level, we considered information at Family level. Additional considerations such as taxa distribution, resemblance, and expert judgment were also applied.

### Trait expression

A fuzzy coding procedure was applied to describe trait expression, scoring each of the taxa analysed depending on the affinity that a taxon displayed with a trait-modality^13,35–37^. Our fuzzy coding used a scoring range from 0–1, with 0 being no affinity and 1 being high affinity to a trait. For example, coding the trait ‘Feeding mode’ for *Amalda novaezelandiae* (Gastropoda), considered that *A. novaezelandiae* is a predatory species, however it also exhibits scavenger feeding, giving a fuzzy coding of 0.5 as predator, and 0.5 as scavenger, completing the full allocation of 1 for the feeding mode trait.

## Data Records

The New Zealand Trait Database (NZTD) and related datasets are available in xlsx-formatted files and can be freely viewed and downloaded from the repository *Figshare*^38^ and National Institute of Water and Atmospheric Research (NIWA) website^39^. Five csv files are available for download: (1) The New Zealand Trait Database of marine benthic invertebrates^38^, (2) the trait information sources^34^, (3) the references by taxa dataset^40^, (4) the full references list^41^, and (5) the full taxa list^18^ used in the NZTD.

### Taxa included

**Fig. 2:**
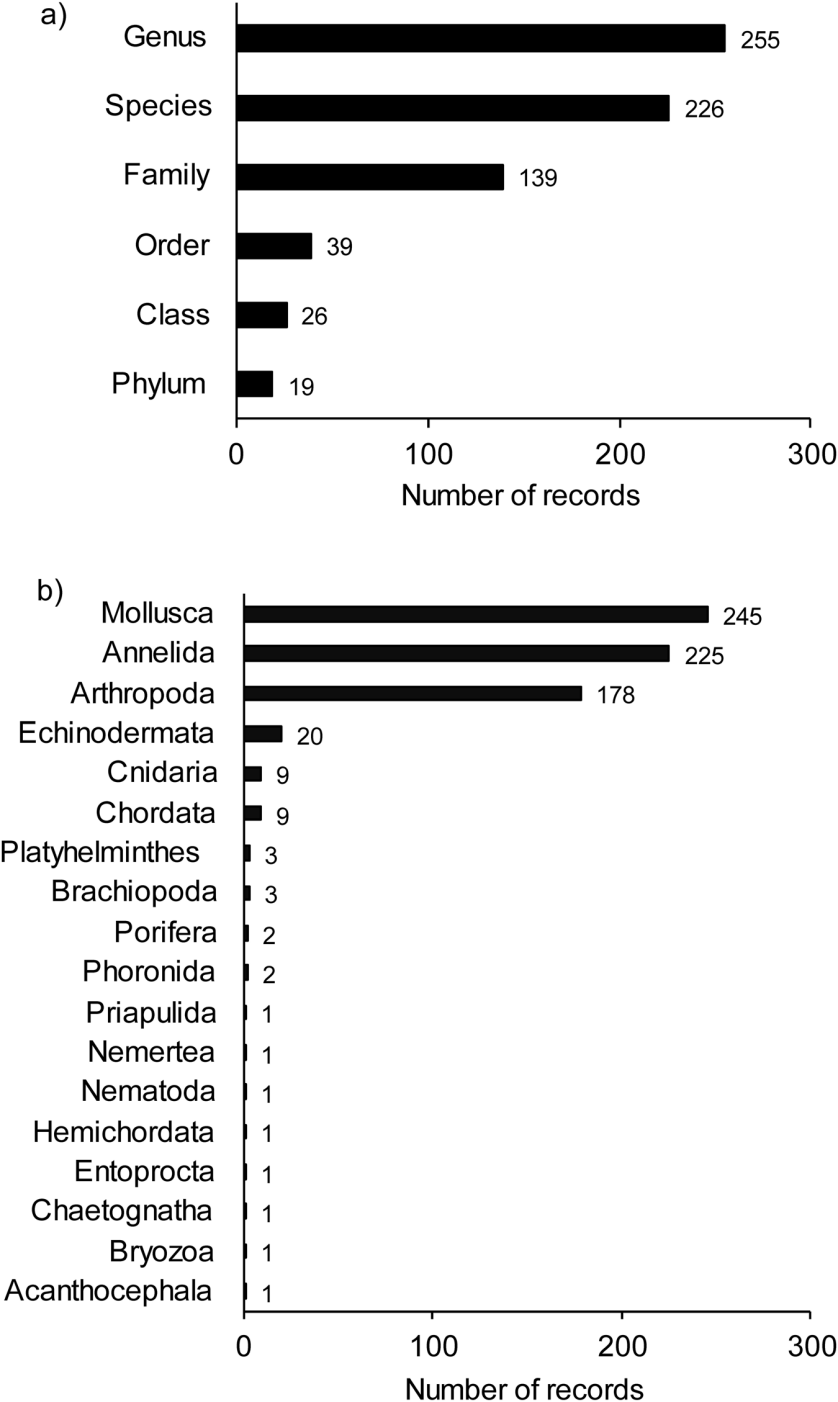
(**a**) Number of taxa assessed at different levels of identification across New Zealand. (**b**) Number of taxa recorded by Phylum across all locations in New Zealand.

In total, we provide trait information for 702 taxa^18^. Different levels of taxonomic identification were assessed, 225 at Species level, 254 at Genus level, 139 at Family level, and the remaining 28 taxa at higher levels (Order, Class, or Phyla; Fig. 2a). The phylum with most records was Mollusca (244 records, 35% of all taxa), followed by Annelida (224 records, 32% of all taxa) and Arthropoda (178 records, 25% of all taxa), with the remaining 8% of taxa belonging to 15 different phyla (Fig. 2b).

### Trait sources

**Fig. 3:**
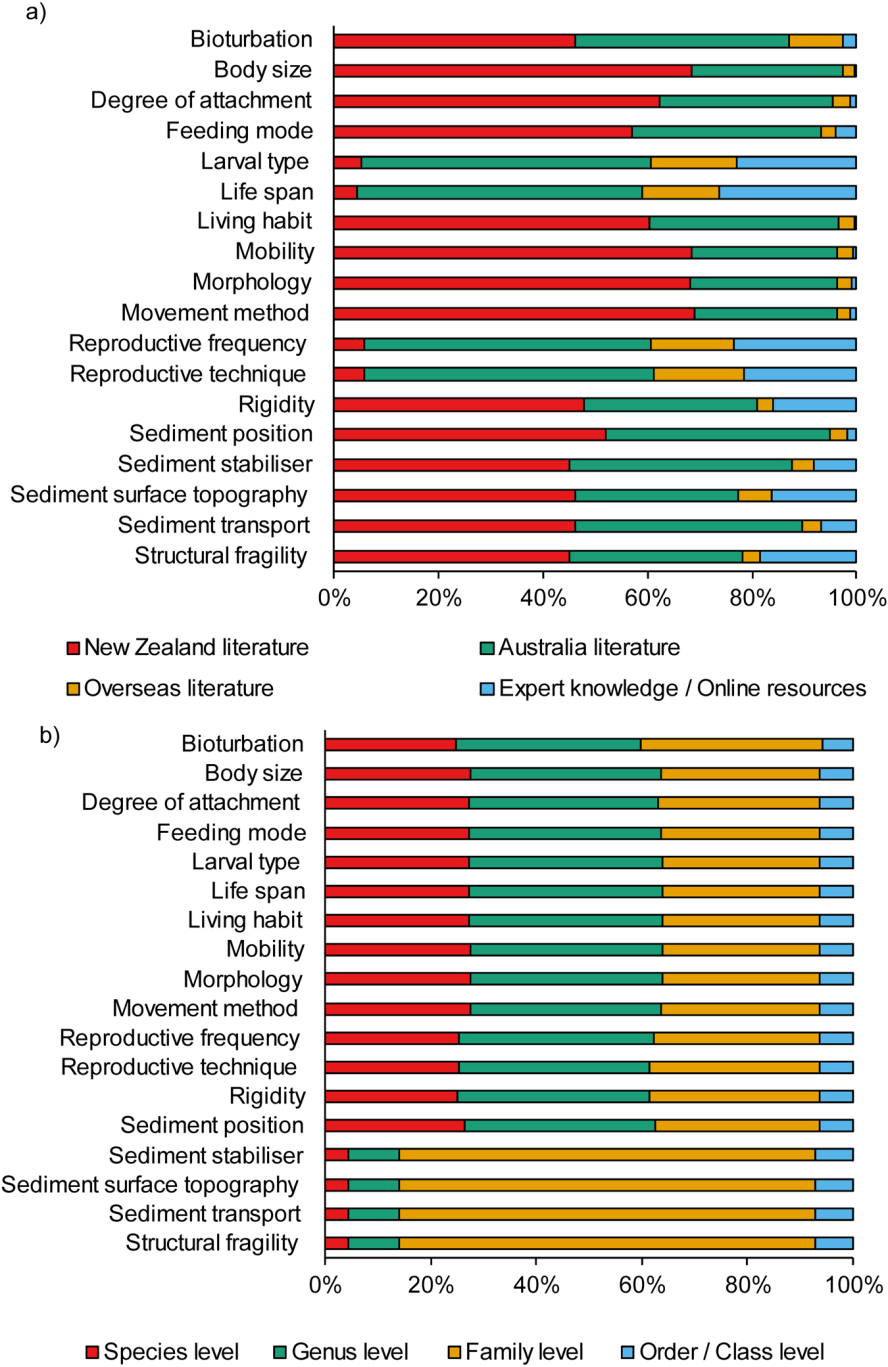
Stacked bar graphs showing (**a**) the cumulative percentage of trait information sources, and (**b**) the cumulative percentage of trait information by taxonomic level.

Trait information was retrieved from several sources and a database containing this information was created for easy interpretation and useability^34^. Including all the traits assessed, 90% of the information was retrieved from primary and secondary sources, which included 45% from New Zealand literature, 39% from Australian literature and 7% from overseas literature. The remaining 9% of information was obtained from reputable resources online and expert knowledge^34^. Yet, we found that the source of trait information differed between types of traits (Fig. 3a). Across taxonomic levels, most of the trait information retrieved was available at the Family (41%), Genus (30%), and Species (21%) levels, with proportionally less at the Order/Class levels (6%; Fig. 3b). It was also evident that the traits larval type, life span, reproductive frequency and technique are less studied for the New Zealand macrobenthic fauna.

## Technical Validation

The New Zealand Trait Database (NZTD) is based on macrobenthic fauna datasets previously validated, used, and published in different articles (e.g.^19–33^,) and official reports, in which macrobenthic fauna were used to understand ecosystem functioning and to evaluate responses to environmental change.

Trait information was compiled using the same approach as for the SAMT database^13^, i.e., the NZTD retrieved information from the most reliable sources available with an accompanying dataset presenting all the references used for each taxon (trait references by taxa^40^; full references list^41^). In addition, the NZTD delivered a detailed dataset showing the taxonomic resolution of the trait information used for each taxa^34^.

## Usage Notes

The New Zealand Trait Database (NZTD) can be freely viewed and downloaded from the repository *Figshare*^38^ and NIWA website^39^. The information allocated on the *Figshare* repository is a static version of the data last reviewed on July 2023, further updates will be released in the same *Figshare* project, but the DOI could be different. A report record will be also included to control further updates. Yet, the datasets available on the NIWA website are dynamically updated.

The NZTD is an ongoing project, with continuous updates and refinements as additional taxa and trait information becomes available. Future updated releases will be published in the same host repositories^38,39^, seeking to keep the structure of NZTD as simple as possible, avoiding complexity, redundancy, and duplication between traits as it expands to include more taxa and traits. As the NZTD evolves, we strongly suggest users to approach the database with awareness of its limitations of available taxonomic and trait-based information, ongoing changes to taxonomic nomenclature, trait information, and trait classification. Future developments may also include an expanded trait database encompassing New Zealand and Australian macrobenthic fauna.

## Data Availability

This research did not use or generate any coding to present the data described in the manuscript.
